# Importance of vitamin D in gastrointestinal health and disease

**DOI:** 10.3389/fnut.2026.1807154

**Published:** 2026-05-18

**Authors:** Samuel G. Sonsalla, Jennifer Armbruster-Lee, Amali E. Samarasinghe

**Affiliations:** 1 Department of Medicine, Division of Allergy Pulmonary and Critical Care, University of Wisconsin-Madison, Madison, WI, United States; 2 Department of Pediatrics, The Ohio State University College of Medicine, Columbus, OH, United States; 3 Divisions of Gastroenterology and Clinical Informatics, Nationwide Children’s Hospital, Columbus, OH, United States; 4 Department of Pediatrics, University of Tennessee Health Science Center, Memphis, TN, United States

**Keywords:** gut-lung axis, IBD, metabolism, supplementation, vitamin D receptor

## Abstract

Since its discovery as an antirachitic agent, vitamin D has been recognized for its importance to health, most namely bone health, prompting food fortification practices that are still in place today. The two forms of vitamin D, ergocalciferol (D_2_) and cholecalciferol (D_3_), are obtained through the diet or synthesized in the skin via ultraviolet-B radiation. Vitamin D is activated by enzymes mainly found in hepatic and renal tissues, to exert downstream effects via the nuclear vitamin D receptor (VDR). VDR is expressed nearly in all tissues, allowing activated vitamin D to have pleiotropic functions. This review focuses on the gastrointestinal tract, with high VDR content and known associations between vitamin D deficiency and disease states. For example, VDR expression within esophageal submucosal glands may influence fibrosis in eosinophilic esophagitis, while in the stomach VDR is associated with malignancy and *Heliocobacter pylori* infection. Intestinal diseases like inflammatory bowel disease, intestinal failure, and irritable bowel syndrome have also been linked to vitamin D deficiency and VDR expression. Although there are studies focused on the impact of genetic polymorphisms in VDR and vitamin D supplementation on intestinal diseases, they remain largely inconclusive at this time. Interest in the gut-lung axis has further prompted the investigation of vitamin D in respiratory conditions like chronic pulmonary obstructive disease, asthma, and *Mycobacterium tuberculosis* infection. While it is clear that vitamin D is important to both the gut and the lungs, there is still a need for further research on the overall role of this important vitamin.

## Introduction

Vitamin D was first discovered as an antirachitic agent and mistakenly described as an amine molecule vital to life. Following conventional alphanumeric naming, vitamin D was the fourth ‘vital amine’ to be discovered and was thus named ‘D’. However, vitamin D is not an amine; it is a fat-soluble sterol that functions as a hormone. Recommendations on dietary intake of vitamin D are based on its established role in bone health (1, 2). Its tissue-specific effects are mediated through the nuclear vitamin D receptor (VDR) which is found in nearly all human nucleated cells, allowing a wide-range of physiological functions (3). Beyond its established role in bone health, VDR activation leads also to transcription of a multitude of metabolic pathways (4). Although causation has not been established, numerous *in vitro* and *in vivo* observational studies report associations between low serum vitamin D levels and diseases across multiple organ systems (5–11), underscoring the potential of vitamin D and its analogs as important catalysts of human health (12). Reflecting this interest, pharmaceutical companies have developed over 3,000 different analogs for vitamin D (13), and the search term “vitamin D” yields over 20,000 publications on PubMed in the past 5 years alone.

While vitamin D’s classical role in calcium metabolism and bone health is well established, its pleiotropic effects on the gastrointestinal (GI) tract are now increasingly recognized. The GI epithelium uniquely interfaces with luminal and systemic vitamin D, positioning it at the intersection of endocrine, immune, and microbial interactions. However, the translation of vitamin D biology into GI-specific pathophysiology remains fragmented. Vitamin D deficiency has been linked to a variety of GI disorders and cancers (14, 15), and supplementation has been shown to improve both symptoms and inflammatory markers in mucosal and functional GI conditions (16–18). Vitamin D deficiency was not associated with increases in VDR mRNA expression, as supplementation increased serum vitamin D levels, but failed to show increase in VDR mRNA in peripheral blood mononuclear cells (PBMCs) (19). The GI tract represents a unique therapeutic target for vitamin D because the intestinal epithelium is exposed from both the luminal and systemic sides and has high expression of VDR. However, the complex pathophysiology of VDR in the gut remains incompletely understood despite its high expression together with that of *CYP27B1* (codes for 1-alpha-hydroxylase that converts inactive vitamin D to calcitriol) in the intestinal epithelial cells.

This narrative review integrates mechanistic and clinical data to vitamin D signaling as a critical regulator of gut epithelial integrity, immune modulation, and fibrosis. We used search terms like ‘vitamin D’, ‘VDR’, ‘gastrointestinal’, ‘intestinal barrier’, and ‘inflammation’ to identify manuscripts, primary and review articles on the topic, based on mechanistic relevance that are aligned with the goals of this review. We examine vitamin D metabolism and the established effects of VDR signaling in the gut, with a focus on clinical vitamin D deficiency in non-cancer related gastrointestinal disorders. We also briefly discuss known functions of vitamin D in the lungs as an extension of the gut-lung axis. By bridging bench findings with patient-centered outcomes, we propose a unifying framework to understand vitamin D’s role across a spectrum of non-malignant GI diseases since understanding of the role of vitamin D in the GI tract is imperative to identifying potential treatment options for a variety of GI disorders.

### Vitamin D nomenclature

**Figure 1:**
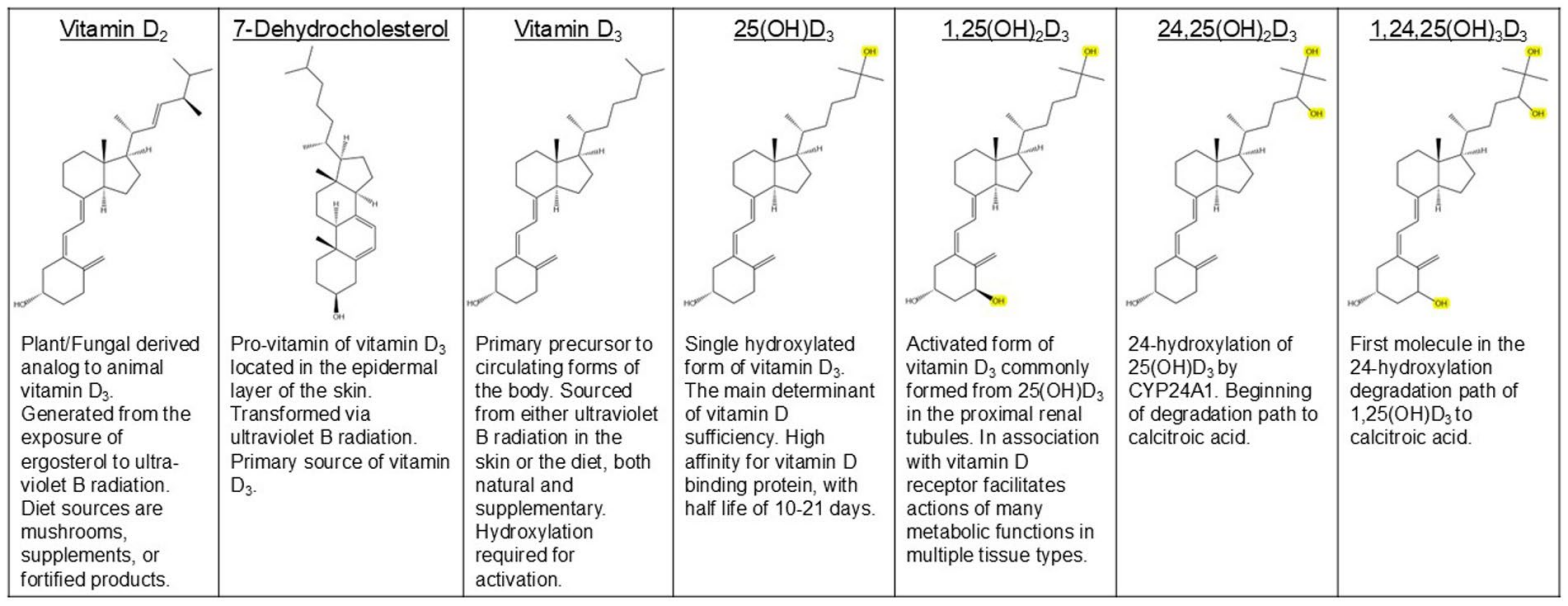
Structures and overview of common metabolites of vitamin D. Hydroxyl groups added in metabolism of vitamin D_3_ are highlighted.

The term ‘vitamin D’ broadly defines various metabolites, requiring further contextual specification. The ‘D’ is a catch-all for metabolites of both ergocalciferol (vitamin D_2_) and cholecalciferol (vitamin D_3_) origins (Figure 1). Therefore, classification via a subscript is necessary to define which form is relevant to the discussion. In this manuscript, ‘vitamin D’ will refer to the cumulative of circulating 25(OH)D and is active metabolite 1,25(OH)_2_D, and a subscript will differentiate the base molecules when only considering one or the other. In clinical settings, total serum 25-hydroxyvitamin D (25(OH)D) plasma levels may be named as the ‘vitamin D’ concentration because the measurement for total serum 25(OH)D is the primary method of determining vitamin D sufficiency (1).

### Sources of vitamin D

**Figure 2:**
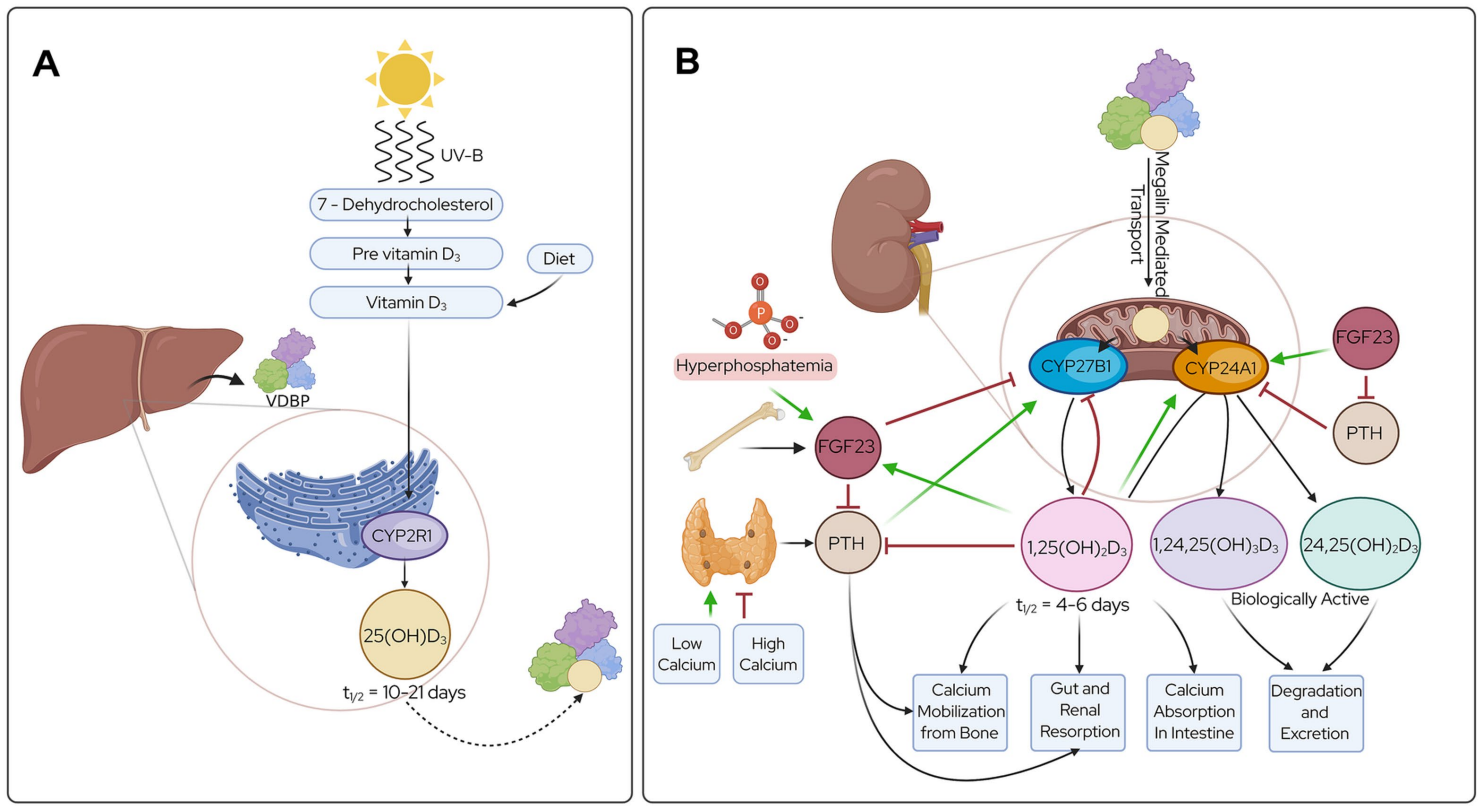
Endocrine metabolic pathway of vitamin D_3_ from the source to an activated state. **(A)** Hydroxylation of vitamin D_3_ by hepatic CYP2R1 to form 25(OH)D_3_, a molecule with high affinity for DBP and relatively long half-life. **(B)** A second hydroxylation of vitamin D_3_ in the kidney by CYP27B1 to produce the active molecule 1,25(OH)_2_D_3_. 1,25(OH)_2_D_3_ regulates the calcium and phosphorus levels in serum through absorption and mobilization of calcium from bone. CYP27B1 is regulated through PTH, FGF23, relative levels of calcium and phosphorus, and product 1,25(OH)_2_D. A separate enzyme CYP24A1 is regulated through similar metabolites to control the degradation pathway of vitamin D_3_ active compounds. Created with BioRender.com.

There are two forms of vitamin D—plant-derived vitamin D_2_ and animal-derived vitamin D_3_. Both are considered pro-hormones since they require hydroxylation to 1,25-dihydroxyvitamin D (1,25(OH)_2_D) (Figure 1) to be biologically active (Figure 2). While both forms of vitamin D can be acquired in the diet through foods like cod liver oil, certain fish, mushrooms, supplements, and fortified products, only vitamin D_3_ can be endogenously synthesized (1, 2).

Vitamin D_3_ is synthesized in the skin when sun ultraviolet (UV)-B radiation converts 7-dehydrocholesterol, a precursor of cholesterol, to pre-vitamin D_3_, further isomerizing to vitamin D_3_ (Figure 2) (2). Studies suggest that 5–30 min of direct sun exposure at least twice per week without sunscreen is usually sufficient to establish adequate vitamin D_3_ synthesis. Time of day, clothing coverage, shade, cloud coverage, sunscreen application, skin melanin content, and latitude all affect vitamin D_3_ synthesis (2, 20, 21). However, because these varying factors impact natural vitamin D_3_ synthesis and global vitamin D deficiency is a major cause of public health concern (22) especially given its importance to bone health, vitamin D is considered an essential dietary vitamin supplement (2, 20, 21, 23).

The discovery of vitamin D as a treatment and cure for rickets in the 1920s led to a sunshine movement in the 1930s–1940s, where people were urged to spend time outside or take daily supplements of cod liver oil (3, 24). During the same time, the importance of fortifying products with vitamin D as a method of preventing rickets gained attention (24). At first, foods such as yeasts, grains, and dairy were irradiated with UV to increase antirachitic properties, a method developed on the knowledge that UV treated oils were found to lower rachitic activity in chickens and rodents (24). Soon after, most milk in the United States and Europe was fortified with vitamin D_2_ to prevent rickets (24). Fortification of other products besides milk, such as soap, shaving cream, beer, and custard around the world was commonplace due to the positive stigma surrounding vitamin D as a miracle drug for chronic illness (2, 24). The global era of fortification ended in the 1950s as children in Great Britain developed varying medical conditions that were tied to the overconsumption of vitamin D, leading to changes in legislature in many European and Asian countries to restrict fortification of products (1, 2, 24). However, fortified foods are still the primary source for the dietary intake of vitamin D in the general population in the United States since few foods naturally contain vitamin D outside of fatty fish (25). Both vitamin D_2_ and D_3_ are available in supplementation forms which can be taken daily or weekly (1, 2). A consensus statement is still lacking regarding the best-suited vitamin D supplement type or frequency is for GI-related disorders.

## Vitamin D absorption and metabolism

Vitamin D_2_ and D_3_ share similar modes of absorption, circulation, metabolism, and biological impacts. Skin synthesis is the dominant source of vitamin D_3_, while gut absorption is secondary, followed by oral intake of supplements. The differences between molecules, such as activation rates and catabolism pathways result from slight structural differences, an additional double bond and methyl group in vitamin D_2_ (Figure 1) (23, 26).

### Intestinal absorption of vitamin D_3_

Under the typical post prandial situation where fatty acids, monoglycerides, and lipids are at high concentration in the intestinal lumen, vitamin D_3_ is primarily absorbed via the incorporation into mixed micelles formed with bile salts and excreted into the lymph through chylomicrons. Transport proteins involved in cholesterol and other fat absorption likely help facilitate absorption of vitamin (23) while some vitamin D_3_ is released by non-chylomicron-dependent pathways directly into portal venous circulation (23). At high intraluminal vitamin D_3_ concentrations or low total intraluminal lipid concentrations, vitamin D_3_ can be absorbed by passive diffusion as well. Although passive diffusion occurs, it is insufficient in the absence of micelle-mediated absorption. The exact mechanism or regulatory pathway by which absorbed vitamin D_3_ enters the lymph or portal venous circulation remains an enigma (23).

### Circulation of vitamin D_3_ and metabolites

Vitamin D_3_ circulates the body in three main forms based on hydroxylation status, vitamin D_3_, calcidiol (25(OH)D_3_), and calcitriol (1,25(OH)D_3_) (Figure 1). Each form of circulating vitamin D_3_ metabolite has variable half-life, activity, and stability. For example, 25(OH)D_3_ has a half-life of approximately 10–21 days while 1,25(OH)_2_D_3_ has a half-life of approximately 4–6 h (Figure 2) (27, 28). The most abundant vitamin D_3_ metabolite in circulation is 25(OH)D_3_. The relative concentration of each metabolite is highly regulated based on physiological need, but these mechanisms are not fully understood. Vitamin D sufficiency is determined by measuring 25(OH)D levels in serum, and a value below 20 ng/mL is considered deficient while 21–29 ng/mL is considered insufficient (1). These serum 25(OH)D thresholds are largely based on skeletal outcomes and may not reflect optimal levels required for mucosal regulation or epithelial function in the gastrointestinal tract. Additionally, environmental and health conditions for each patient, such as daily sunlight exposure and age, influence desired ranges and supplementation amounts for vitamin D. Additionally, varied methods for measuring blood 25(OH)D levels can result in differential values in repetition or across patients, making it difficult to create definitive cutoffs for deficiency (1).

As a sterol, vitamin D_3_ is lipophilic and freely passes through cell membranes. However, most circulating vitamin D_3_ and metabolites are bound to the vitamin D-binding protein (VDBP), a glycoprotein synthesized primarily in the liver with high affinity for vitamin D_3_ metabolites (23). The half-life of each circulating form of vitamin D_3_ metabolite is dependent upon its affinity with VDBP. The 100-fold lower dissociation constant of 25(OH)D_3_ compared to that of vitamin D_3_ and 1,25(OH)_2_D_3_ signifies the higher affinity for VDPB and the far greater half-life expressed previously (29). When 25(OH)D_3_ is bound to the VDBP, uptake into cells occurs through megalin-mediated endocytic transport primarily in the kidneys but also in select other cells like the parathyroid glands (29). Megalin-mediated transport in the kidneys is key to the renal resorption of 25(OH)D_3_ in the tubular epithelial cells to avoid loss of 25(OH)D_3_ to urine and production of the active form 1,25(OH)_2_D_3_ (Figure 2) (30). The difference in cellular transport provides a level of tissue specificity to vitamin D_3_ bound to VDBP beyond simple diffusion. The utility of the megalin-mediated endocytosis for the delivery of 25(OH)D_3_ into the parathyroid glands shows the importance of vitamin D_3_ on the endocrine system and its role in the suppressing parathyroid hormone (PTH) production (29, 30) (Figure 2).

### Vitamin D_3_ metabolism

There are three main hydroxylation steps involved in the metabolism of vitamin D_3_ that occur via enzymes of the cytochrome P450 system (Figure 2). CYP2R1, also called 25-hydroxylase, is on the endoplasmic reticulum of cells and performs the 25-hydroxylation step leading to 25(OH)D_3_. CYP27B1, also called 1α-hydroxylase, and CYP24A1, also called 24-hydroxylase, are mitochondrial enzymes involved in activation and degradation of 25(OH)D_3_ to 1,25(OH)_2_D_3_ and 24,25(OH)_2_D_3_, respectively.

Vitamin D_3_ is activated via endocrine or autocrine pathways. The best characterized metabolic pathway is the endocrine pathway accounting for vitamin D_3_ involvement in calcium, phosphorus, and bone homeostasis (Figure 2). CYP2R1 exerts effects in the liver. The resultant circulating 25(OH)D_3_-VDBP is taken into the proximal tubule of the nephron via megalin-mediated uptake where CYP27B1 activates 25(OH)D_3_ to 1,25(OH)_2_D_3_. *CYP27B1* is upregulated by PTH and inhibited by fibroblast growth factor (FGF)-23 and 1,25(OH)_2_D_3_. The expression of PTH and FGF-23 is regulated by serum calcium and phosphorus levels, respectively. Elevated 1,25(OH)_2_D_3_ stimulates intestinal calcium absorption and together with elevated PTH, enhances calcium mobilization from the bone, and gut and renal resorption, correcting hypocalcemia. *CYP24A1* is upregulated by FGF-23, elevated by 1,25(OH)_2_D_3_, and inhibited by PTH. CYP24A1 hydroxylates 25(OH)D_3_ and 1,25(OH)_2_D_3_ to 24,25(OH)D_3_ and 1,24,25(OH)_2_D_3_ (Figure 1) of which can be further degraded by CYP24A1 to calcitroic acid for excretion primarily in bile, as well as feces and urine (Figure 2) (21, 26, 29). Given the central role of the liver in vitamin D metabolism, liver disease is frequently associated with vitamin D deficiency. This is attributed to impaired hepatic 25-hydroxylation, reduced VDBP production, advanced disease states, and impaired absorption of vitamin D (31).

More recently, it has been discovered that vitamin D_3_ can passively diffuse into cells and be locally activated via CYP27B1, exerting effect by modulating VDR in an intracrine, autocrine, or paracrine fashion (29, 32). CYP27B1 has been found in colonic epithelium and is present in macrophages that span the GI tract. Local activation of this enzyme may require a co-factor such as transforming growth factor (TGF)-β, or an increase in toll like receptors (TLR) secondary to infection, epithelial injury, or inflammation (33, 34).

## Effects of vitamin D receptor

VDR is a nuclear receptor expressed on numerous tissue types but is found most abundantly in the GI tract (35). The gene responsible for VDR expression is located on chromosome 12q13.11 (36), and mutations of this gene are known to increase risk of various diseases, including inflammatory bowel disease (IBD) (37–39). Importantly, VDR expression is not only a component of the human genome, but it is also important in regulating the gut microbiome and its metabolites (40).

### Vitamin D receptor structure and signaling

VDR is composed of multiple structural protein domains. Included are the N-terminus domain (A/B), DNA binding domain (DBD, C), a hinge region (D), and ligand binding domain (LBD) prominent in the C-terminus (E) (41–44). Zinc finger structures within the DBD are responsible for recognition of vitamin D response elements (VDREs) within the genome when associated with retinoid X receptor (RXR) (41). The LBD is located internally in folded VDR, composed of 12 α-helices compacted into a three-layered structure and a three-stranded β-sheet, responsible for specific ligand binding and coactivator association potential (41, 43–45). Recently, the investigation of non-genomic rapid signaling cascades caused through VDR activation led to potential of two overlapping ligand binding pockets being present within VDR, the genomic pocket (VDR-GP) and alternative pocket (VDR-AP) (41–43, 46). This has brought about the conformational ensemble model, where both apo-VDR LBD and natural ligands, like 1,25(OH)_2_D_3_, are present in multiple structural configurations, allowing for varied ligand binding to pockets, and therefore differences in downstream effects (41, 42, 46). Based upon the orientation of the ligand and the pocket the ligand binds, the result would be either longer genomic response or rapid signaling, respectively.

Once 1,25(OH)_2_D_3_ binds to the VDR, it interacts with a variety of other proteins including co-activators, co-repressors, and the RXR which together form the VDR complex. The active VDR complex, from the genomic pocket, regulates multiple genes that typically contain VDREs composed of direct repeat half-element hexanucleotides with three nucleotide spacers (DR3) (41, 47). Regulation from epigenetic changes usually takes hours or days (41). These genes code for proteins involved in bone and renal metabolism, LRP5, RANKL, Klotho, FGF23, and PTH, mineral metabolism, TRPV6 (calcium), cell proliferation, apoptosis, and differentiation, contributing to its multitude of tissue-specific effects (13, 41). The VDR-GP is approximately 56% filled when bound to 1,25(OH)_2_D_3_, therefore, pharmaceutical analogs of 1,25(OH)_2_D_3_ can activate the VDR complex, modulating its shape and affinity for co-activators and repressors, ultimately impacting downstream effects of VDR signaling (13, 44). Also, other ligands like lithocholic acid can bind, although different to 1,25(OH)_2_D_3_, to result in modified downstream signaling (43).

Activation of VDR for rapid signaling pathways is generally initiated with membrane VDR associated with caveolae (41, 42, 45, 46). It is thought that binding ligands to the VDR-AP initiates rapid responses through activation of common signaling pathways such as MAP-Kinase and PI3K, leading to faster signaling for biological responses such as opening of calcium and chloride ion channels (41, 45). As activation of signaling pathways may result in genomic response, cross talk between VDR rapid responses and genomic responses occurs, further complicating the complex signaling system. The VDR-AP has been shown to preferentially bind to 1,25(OH)_2_D analogs that possess the 6-*s-cis*, planar orientation, rather than 6-*s-trans*, boat orientation (41, 42, 45, 46).

### Vitamin D receptor and the gut

The importance of the intestinal VDR in the regulation of calcium and phosphate absorption is well recognized (41). Murine studies have demonstrated that VDR signaling plays an important role in maintaining a healthy gut barrier. VDR functions as an anti-inflammatory and anti-fibrotic mediator that regulates a healthy microbiota and detoxifies substances such as lithocholic acid from the bowel, preventing formation of colorectal cancer (40, 48–57). When guinea pig ileum *in vitro* are co-treated with calcium and 1,25(OH)_2_D_3_, intestinal motility and contraction improve (58). Clinical studies of VDR expression continue to expand knowledge of the importance of vitamin D in the GI tract and in one health.

A recurring theme across GI diseases is epithelial dysfunction, which ranges from impaired barrier integrity in IBD to tissue remodeling in eosinophilic esophagitis (EoE). VDR signaling intersects these processes at multiple levels, including suppression of pro-fibrotic pathways (e.g., TGF-β/Smad), regulation of epithelial-mesenchymal transition, and modulation of innate immune sensors (59). The capacity of intestinal epithelial cells to locally activate 25(OH)D_3_ via CYP27B1, particularly to protect against inflammation (60), further supports a model of mucosal regulation. Understanding how VDR activation modifies epithelial and immune cell crosstalk, and the microbiome may identify novel therapeutic targets beyond systemic supplementation alone. Although VDR signaling shows potential for multitude of therapeutic responses within the gut, ultimately the current understanding of VDR impact is still unclear due to the dearth of clinical human data.

## Clinical studies of vitamin D and the GI tract

### Vitamin D and the esophagus

Cells of the submucosal glands and ducts deep to the squamous mucosa of the normal esophagus contain VDR (61). While patients with VDR associated polymorphisms have decreased VDR expression in the esophagus (62), the clinical significance is yet to be determined.

There are no current studies to suggest serum 25(OH)D deficiency is associated with increased risk of esophageal motility disorders or gastroesophageal reflux disease (GERD). A single case report of a rare multisystem disorder, alacrima, achalasia, adrenal failure, progressive neurodegenerative disorder, caused by *AAAS* gene mutation, demonstrated low bone mineral density (BMD) and serum 25(OH)D levels as an under-recognized symptom (63). Dietary intake of vitamin D in pediatric patients with GERD was recently found to be below recommended dietary intake for age, but no serum deficiency was found in either group when measured by Clinical and Translational Research Centers Core Laboratory Services (64).

Serum 25(OH)D levels measured in EoE patients have led to the conclusion that these patients may be both vitamin D sufficient (64) or insufficient (65). EoE is a progressive fibrostenotic disease presenting various age dependent symptoms from vomiting in children to food impaction in adults (66). Although pediatric EoE patients may consume less vitamin D (64), possible long-term effects on esophageal remodeling such as progression to fibrosis remains unknown. Currently, there is insufficient evidence to establish that low levels of 25(OH)D or reduced VDR contribute to EoE pathogenesis. The VDR, however, is known to negatively regulate TGF-β signaling (67) which is central to the fibrotic remodeling characteristic of progressive EoE (68–70). VDR is highly expressed in the esophageal epithelium, underscoring its potential role as a transcription factor in this tissue (71). In an IL-13-induced model of esophageal inflammation, *in vitro* supplementation of 1,25(OH)_2_D_3_ reversed the expression of several IL-13-responsive genes, including TGF-β receptor (TGFBR1), a gene linked to the epithelial-mesenchymal transition (EMT) and fibrosis (71). Previous murine and human studies treating vitamin D deficiency were able to induce VDR expression and decrease activity of TGF-β in multiple cell types (48, 66). These findings support a model in which the vitamin D-VDR signaling may modulate fibrotic and inflammatory pathways relevant to EoE, including potential effects on eosinophil function.

### Vitamin D and the stomach

Beyond its role in immunity and host defense, vitamin D may influence gastric function through effects on epithelial integrity, neuroendocrine signaling, and nutrient absorption. VDR is located in the cytoplasm and perinuclear regions of gastric epithelial and mucosal cells (72). Although *VDR* is expressed in lower rates in gastric malignant tissues compared to normal or premalignant tissue (72), no strong clinical correlation between VDR concentration and non-carcinomic conditions in gastric tissue has been described to date. Low levels of serum 25(OH)D_3_ has been associated with autoimmune gastritis (73) and in *Helicobacter pylori* infection with treatment failures (74, 75). *VDR* expression is increased in *H. pylori* infected gastric mucosa and correlates with inflammation and increased cathelicidin antimicrobial peptide (CAMP) mRNA, suggesting that VDR signaling is important in gut host defense mechanisms (76).

Gastric physiology and drug-nutrient interactions may also be impacted by vitamin D. Growing evidence indicates a link between impaired gastric motility and low serum 25(OH)D levels. Higher serum 25(OH)D was associated with improved gastric emptying rate in gastroparesis patients suggesting a possible role for vitamin D in the function of the enteric nervous system (77). Additionally, prolonged proton pump inhibitor (PPI) use may adversely impact BMD (78) and, potentially, vitamin D metabolism. While some studies report reduced 25(OH)D levels in chronic PPI users, others show no change (79). Supplementation with 1,25(OH)D_3_ concurrently with PPIs has not been shown to have an effect on BMD (80) despite its ability to increase calcium absorption. Therefore, the need for further research to clarify the role of vitamin D on overall gastric health especially in the context of PPI use is necessary.

### Vitamin D and the small intestine/colon

In contrast to the esophagus and stomach, the intestines are the most extensively studied for VDR function in GI health and disease. VDR is located in cells throughout the small intestine and colon in high concentration (32) re-iterating its key role in GI physiology. The *VDR*, located on chromosome 12q13.11, has been linked to IBD on genome wide association studies (GWAS) (54, 81). Four single nucleotide polymorphisms (SNPs) identified and named for the restriction enzyme that recognizes the polymorphism (ApaI, BsmI, TaqI, and FokI) are found within the *VDR*. Although there is mixed evidence for these SNPs on the pathogenesis of Crohn’s disease (CD) or ulcerative colitis (UC), a meta-analysis across ethnic groups found that TaqI tt genotype increased the risk for CD compared to TT in Europeans, and the ApaI ‘a’ allele was protective (81). In contrast, the FokI variant is associated with increased risk of UC but not CD in Asians (81). The Nurses’ Health Study, in which a cohort of 72,719 female registered nurses were followed prospectively using biennial questionnaires, showed that higher serum 25(OH)D levels correlated with a reduced risk of developing CD but not UC (82). Low serum 25(OH)D levels are consistently associated with higher symptom index scores and also elevated fecal calprotectin levels in both CD and UC even when patients are in clinical remission (54, 83, 84). Vitamin D deficiency is a recognized risk factor for decreased BMD in IBD patients (85). In a prospective randomized double-blinded controlled clinical trial in pediatric IBD patients aged 5 to 18 years, introduction of 2,000 IU/day of vitamin D_3_ alongside other IBD treatment led to significant reductions in pro-inflammatory markers, IL-12, IL-17, IL-23, and TNF-α, as well as improved quality of life with no observed adverse effects (86). However, clinical benefits of vitamin D supplementation were only associated with vitamin D deficient patients (86). Normalization of plasma 25(OH)D levels via supplementation in CD patients is associated with a reduced risk of IBD-related surgery but not hospitalizations (17). Similarly, increased UV exposure was protective against CD inpatient surgery (87).

Intestinal failure resulting from complications like short bowel syndrome (SBS) lead to an insufficient absorption of nutrients, water, vitamins, and electrolytes by the intestine. To overcome this inadequacy, parenteral nutrition (PN) is utilized to avoid improper absorption in the intestines (88). Clinically, there is a high prevalence of hypovitaminosis D in patients with intestinal failure with one study documenting up to 75% of their patient cohort as either deficient or insufficient (89, 90). This is thought to be related to poor absorption and chronic illness. These patients are at high risk for metabolic bone disease even after weaning off parenteral nutrition (88). No studies on *VDR* expression in this patient population have been published, presenting a significant gap in knowledge. However, in a recent rodent SBS study, the expression of yes-associated protein (YAP) of the Hippo pathway, responsible for regulating proliferation, apoptosis, and homeostasis, was associated with the expression of VDR protein in the small intestine (91). In both *in vitro* and *in vivo* trials, the introduction of propionate resulted in higher expression of VDR through YAP activation (91). Further research is required to determine the effects of increased expression and levels of resultant intestinal adaptation.

Pediatric IBS patients have lower serum 25(OH)D levels compared to healthy children (92), while in adults, two randomized, double blind, placebo-controlled trials reported decreased IBS symptoms and improved quality of life with vitamin D_3_ supplementation (16, 18). Although it appears that vitamin D affects IBS symptomatology, specific mechanisms that link VDR signaling to IBS remain unclear. A proposed mechanism suggests that VDR regulates the activity of tryptophan hydroxylase (TPH) which acts as the rate-limiting enzyme in serotonin synthesis (93). Serotonin is a neurotransmitter that plays an important role in gut motility, secretion, and sensation, which are all disrupted in IBS. Additionally, its synthesis in the brain is impacted by vitamin D sufficiency (94) implicating vitamin D in the gut-brain axis.

### Impact of vitamin D on gut microbiome

An important pillar of the gut is the large and diverse microbiome which contributes to nutrition, immune regulation, epithelial integrity, and overall health. Dysbiosis of a healthy microbial community has been associated with a range of GI diseases. The established role of vitamin D in gut physiology suggests that vitamin D may influence the composition and function of the gut microbiome, although its precise effects remain incompletely defined.

Previous studies have associated vitamin D deficiencies, VDR polymorphisms, and vitamin D intake with changes in microbial composition in the gut and stool (95). Vitamin D is known to bind and activate gut immune cells, particularly macrophages, enhancing anti-microbial peptide production and phagocytic activity (95, 96). Vitamin D can also indirectly shape the microbiome by maintaining the gut epithelial barrier integrity through tight junction protein regulation, restricting microbial translocation and unlicensed interactions with host cells (95, 96). Interestingly, some bacteria have been reported to express homologs of enzymes involved in vitamin D metabolism such as CYP2R1, CYP27A1, and CYP27B1, implying differences in vitamin D metabolism based on bacterial presence (95). Considering the broad and complex effects of vitamin D on the gut microbiome, further studies are needed to define these interactions and their context-dependent contributions to gut health and disease.

### Vitamin D and gut-lung axis

Vitamin D is a mucosal immunoregulator that operates across barrier tissues, and the gut-lung axis represents a biologically relevant system when shared VDR-dependent pathways including epithelial defense, antimicrobial peptides, and immunomodulation may coordinate organ-level responses. Since both intestinal and lung structures are derived from the endoderm in fetal development, and share immunological features (97), vitamin D signaling may represent a conserved regulatory pathway across these barrier tissues. The bi-directional connection between these tissues, in which associations in disease, dysbiosis, and infection may have resulting impacts on one another (97), signifies the potential impact vitamin D may have in the lungs. Although multiple gut-organ axes have been described, we focus on the gut-lung axis due to the substantial body of work linking vitamin D signaling to pulmonary epithelial cell function and immunity. Human tracheobronchial epithelial cells (hTBEs) constitutively express CYP27B1 mRNA and can produce 1,25(OH)D_3_ when exposed to 25(OH)D_3_, leading to microenvironments with higher levels of active 1,25(OH)_2_D_3_ in the lungs (98). The increased concentrations of 1,25(OH)_2_D_3_ within the lung epithelium can cause autocrine downstream effects through VDR since the expression of VDR-dependent *CYP24A1*, *CAMP*, and *CD14* were shown in higher amounts in epithelial cells exposed to either 25(OH)D_3_ or 1,25(OH)_2_D_3_ (98). The impact of Vitamin D on pulmonary diseases and infections is still not fully understood, but current studies show conflicting results. The lung vitamin D and OmegA-3 Trial (VITAL), a United States nationwide study investigating the effects of vitamin D and fish oil (Omega-3) on pulmonary morbidity, primarily found vitamin D_3_ supplementation to not improve chronic obstruction pulmonary disease (COPD) exacerbation rates or decline in lung function (99). A secondary result similarly found that supplementation left asthma exacerbations and asthma control unchanged (99). Vitamin D_3_ supplementation did not increase time to the next exacerbation (100) or reduce serum levels of total IgE or IgE to common allergens in asthmatic children (101). A separate adult study found significant improvement of lung function (FEV_1_:FVC ratio) in asthmatics over a 12-week period (102). In *Mycobacterium tuberculosis* (Mtb) infection, the supplementation of vitamin D_3_ did not reduce the risk of infection in children (103, 104) but could improve fever and cough symptoms after starting Mtb treatment (105). In adults, similar supplementation of vitamin D_3_ resulted in no impact on sputum Mtb culture conversion (106). Recently, lower 25(OH)D levels, below 15 nmol/L, was associated with significantly higher hazard ratio for respiratory tract infection (RTI) hospitalization compared to higher levels, above 75 nmol/L (107). However, measurements of 25(OH)D were acquired from participants at baseline, without seasonal consideration, and RTIs were only counted based on hospitalization records (107). Overall, these observations reinforce the need to understand vitamin D signaling in a tissue-specific context, particularly in the GI tract where local activation and VDR expression is highly enriched, providing a context for broader interpretation across mucosal organs.

## Research priorities and conclusion

Moving forward, mechanistic studies to clarify the spatial and temporal dynamics of VDR expression and signaling in inflamed versus non-inflamed mucosa are needed to understand its function for further therapeutic implications. The field lacks longitudinal human data linking serum 25(OH)D levels, mucosal VDR activation, and disease progression or treatment response. In addition, studies that incorporate VDR polymorphisms or gut-specific transcriptomic changes may uncover subgroups that derive maximal benefit from vitamin D therapy. Importantly, future trials should differentiate between systemic and luminal delivery routes, particularly in diseases like IBD or intestinal failure where epithelial exposure to vitamin D may be key.

**Figure 3:**
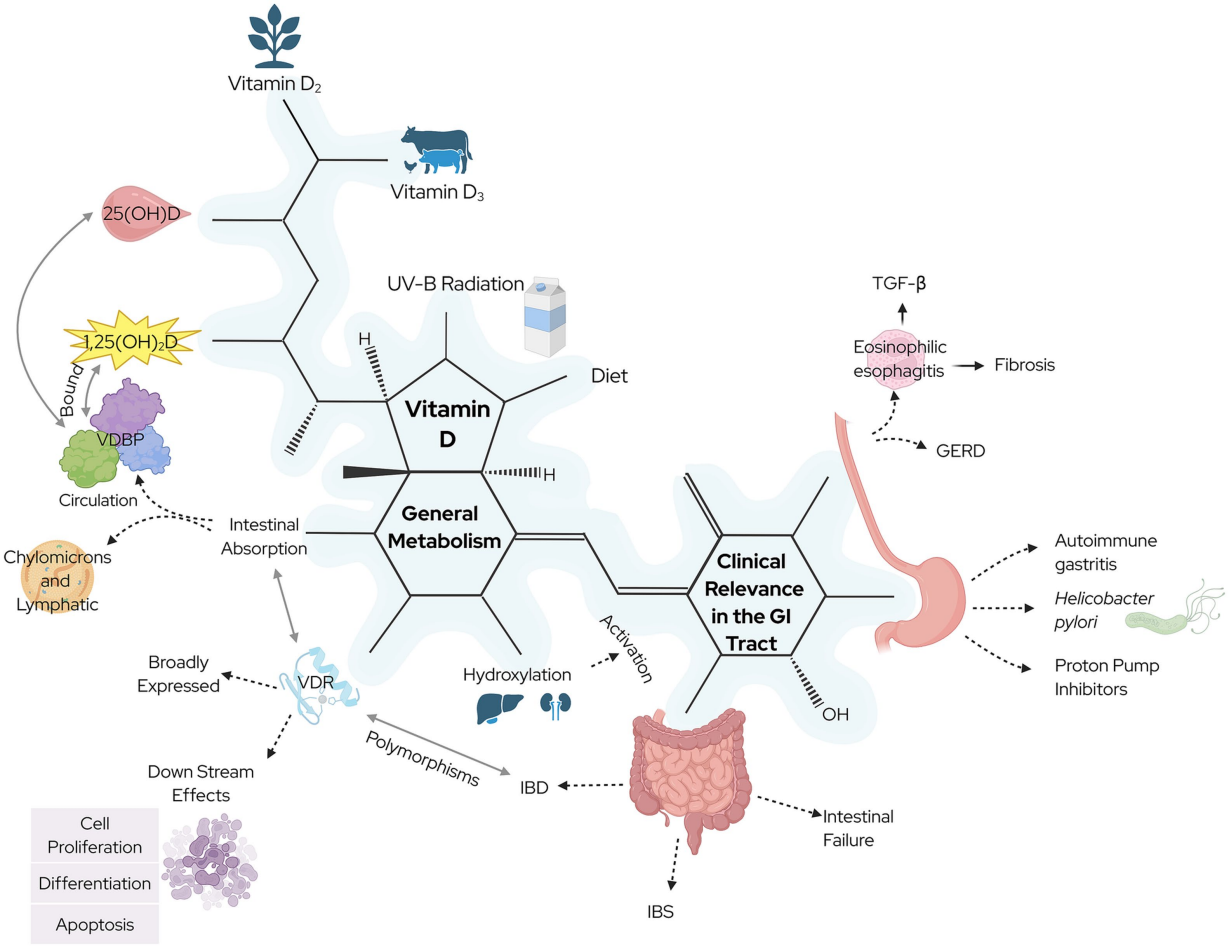
An overview of vitamin D in its history, sources, metabolism, and current clinical research in non-carcinogenic diseases of the gastrointestinal system. Created with BioRender.com.

The evidence reviewed here supports a broader view of vitamin D as a context-dependent immunoregulatory and epithelial-modulating agent in the GI tract (Figure 3). While serum 25(OH)D status is a convenient biomarker, its relationship to mucosal VDR activity remains inadequately defined. Clinicians and researchers must shift toward a model that incorporates tissue-specific vitamin D signaling, local activation, and downstream functional outcomes. As a result, personalized strategies like tailoring vitamin D delivery, dosing, and formulations based on disease subtype, genetic background, and mucosal VDR responsiveness may offer a path forward in optimizing GI health.
